# Early-Onset Neonatal Sepsis: Clinical System Involvement and Maternal–Neonatal Risk Profiles in a Retrospective Cohort Study

**DOI:** 10.3390/children13050639

**Published:** 2026-05-03

**Authors:** Anna Damatopoulou, Michail Matalliotakis, Fani Ladomenou, Christina Thomou, Marina Koropouli, Maria Polychronaki

**Affiliations:** 1 Department of Obstetrics and Gynecology, Venizeleio General Hospital of Heraklion, 71409 Heraklion, Greece; anna.damatopoulou@outlook.com (A.D.); mihalismat@hotmail.com (M.M.); 2 Department of Obstetrics and Gynecology, Alexandra General Hospital, 11528 Athens, Greece; 3 Pediatric Infectious Diseases Unit, Department of Pediatrics, Medical School, University of Ioannina, 45110 Ioannina, Greece; 4 Department of Neonatology and Neonatal Intensive Care Unit, University Hospital of Heraklion, 71500 Heraklion, Greece; cthomou@yahoo.gr; 5 Neonatal Intensive Care Unit, Venizeleio General Hospital of Heraklion, 71409 Heraklion, Greece; koropouli.mar@gmail.com (M.K.); polichronaki.mp@gmail.com (M.P.)

**Keywords:** early-onset neonatal sepsis, clinical system involvement, risk factors

## Abstract

**Highlights:**

**What are the main findings?**

- Assessment of early clinical course and evolution of clinical system involvement throughout the first days of life.
- Identification of early risk profiles contributing to advanced sepsis at presentation, persistent sepsis at Day 7, and mortality.

**What is the implication of the main findings?**

- Noting changes in clinical presentation is essential as infection evolves throughout the primary days of a neonate’s life, thus avoiding unnecessary antibiotic exposure.
- Identifying high-risk neonates is key to optimizing clinical diagnostic and treatment plans.

**Abstract:**

Background/Objectives: Neonatal sepsis remains a major contributor to neonatal morbidity and mortality worldwide, yet diagnostic uncertainty and heterogeneous clinical presentation continue to challenge early recognition and management. Early-onset sepsis (EOS), typically arising within the first 72 h of life, is strongly influenced by maternal and perinatal factors. Limited data exist on the temporal evolution of clinical system involvement during the first week of life. This study aimed to identify the predominant clinical systems involved in preterm and term neonates with suspected or confirmed sepsis and to determine maternal and neonatal risk factors associated with early disease severity, persistent sepsis, and adverse outcomes. Methods: A total of 297 neonates met the inclusion criteria. Most infants (99.3%) were admitted before 72 h of life. Clinical system involvement was recorded daily, and maternal–neonatal risk factors were analyzed to identify predictors of advanced sepsis at presentation, persistent sepsis at Day 7, and mortality. Results: Respiratory involvement was the predominant clinical system affected on Day 1 (57.2%) and remained common through Day 3. CNS, gastrointestinal, and skin involvement were infrequent. Lower gestational age (*p* = 0.035) and prolonged rupture of membranes >18 h (*p* = 0.043) independently predicted sepsis at Day 1. Advanced sepsis at admission was associated with lower birth weight, lower gestational age, older maternal age, and absence of intrapartum antibiotics (all *p* ≤ 0.001). Persistent sepsis at Day 7 was linked to prematurity (*p* = 0.008), higher mortality (*p* < 0.001), and prolonged hospitalization (*p* = 0.001). Conclusions: Respiratory involvement was the most common clinical system affected in neonates with EOS. Prematurity, low birth weight, prolonged rupture of membranes, and maternal intrapartum infection significantly increased the risk of severe disease. Understanding the evolution of clinical system involvement during the first days of life may support more precise risk stratification and reduce unnecessary antibiotic exposure.

## 1. Introduction

Neonatal sepsis remains a major global challenge for healthcare systems, contributing substantially to neonatal morbidity and mortality [1,2,3,4]. It is typically categorized as early-onset sepsis (EOS), occurring within the first 72 h of life and primarily resulting from vertical maternal transmission, or late-onset sepsis (LOS), occurring after 72 h of life and usually reflecting nosocomial or community-acquired infection. The Global Burden of Disease (GBD) Study estimates approximately 1.3 million annual cases of neonatal sepsis worldwide, with global deaths ranging between 400.000 and 700.000 each year [5,6].

Defining neonatal sepsis remains challenging, as many studies highlight the lack of a universally accepted diagnostic standard. The most commonly used definition includes the presence of clinical signs of infection combined with a positive blood culture and/or positive cultures from sterile body fluids such as cerebrospinal fluid, urine, or peritoneal fluid. Suspected sepsis is generally defined as clinical signs of infection accompanied by a positive sepsis screen [4,7,8,9,10]. However, blood cultures may yield false-positive or false-negative results, and clinical signs alone lack diagnostic specificity [7]. This diagnostic uncertainty has contributed to widespread empirical antibiotic use, which is associated with both short- and long-term adverse outcomes in childhood, including asthma, obesity, and inflammatory bowel disease [11,12].

The majority of neonatal sepsis cases occur in premature infants (<37 weeks of gestation) and in very low birth weight (VLBW) neonates (<1500 g) [6,7,8,9,13,14]. Established risk factors include premature rupture of membranes (PROM), chorioamnionitis, intrapartum fever, meconium-stained amniotic fluid, and the use of central venous catheters in the Neonatal Intensive Care Unit (NICU) [4,14,15]. In developed countries, *Group B Streptococcus* (*GBS*) and *Escherichia coli* remain the predominant pathogens responsible for EOS. Universal antenatal GBS screening, recommended by the American College of Obstetricians and Gynecologists (ACOG) between 36 0/7 and 37 6/7 weeks of gestation, has significantly reduced the incidence of *GBS*-related EOS [16]. Nevertheless, *GBS* continues to contribute to neonatal morbidity and mortality in the United States and other high-income settings [9,12,16].

Management strategies differ between EOS and LOS due to variation in their underlying pathogens. Rising antimicrobial resistance has led to deviations from the recommended treatment protocols, underscoring the need for robust antibiotic stewardship [17]. The introduction of the Sepsis Risk Calculator (SRC) for EOS has been associated with reduced antibiotic exposure and improved clinical decision-making [11]. The American Academy of Pediatrics now recommends the use of the SRC for managing neonates ≥34 weeks of gestation [12]. However, recent evidence highlights important limitations of the SRC, including the potential for delayed treatment initiation in high-risk neonates, reinforcing the need for careful clinical monitoring and contextualized risk assessment [18].

During the study period (2016–2019), the SRC was not implemented in our NICU; therefore, clinical evaluation relied on established maternal and neonatal risk factors and daily bedside assessment. Despite extensive literature on EOS risk factors, limited data exist on how clinical system involvement evolves during the first week of life, particularly when daily structured assessments are used. Furthermore, terms such as “incipient”, “advanced”, and “persistent” sepsis are inconsistently applied across studies, complicating comparisons and highlighting the need for clearer operational definitions.

This retrospective cohort study analyzes prospectively collected daily clinical data to: (1) characterize the pattern of clinical system involvement during the first week of life in neonates evaluated for EOS, and (2) identify maternal and neonatal factors associated with early disease severity, persistent sepsis, and adverse outcomes. By mapping the temporal evolution of clinical signs rather than relying solely on admission findings, this study provides novel insight into early disease trajectories in EOS.

## 2. Materials and Methods

### 2.1. Study Design and Setting

This retrospective observational study was conducted in the Level II NICU of Venizeleio General Hospital of Heraklion, Crete, and included all neonates managed between January 2016 and December 2019. Although the analysis was retrospective, all clinical data were recorded prospectively using a structured daily questionnaire completed by attending neonatologists as part of routine clinical care. According to national census data, 25,377 live births occurred on the island of Crete during the study period, distributed among the three NICUs operating on the island.

### 2.2. Study Population

A total of 458 neonates were diagnosed with suspected or confirmed sepsis in our NICU during the study period. Of these, 297 neonates met the inclusion criteria, (1) admission within the first 7 days of life, consistent with the EOS definition applied during the study period, and (2) availability of complete daily clinical documentation. Neonates with incomplete documentation or those transferred after the first week of life were excluded. We acknowledge that exclusion due to missing documentation may introduce selection bias; however, the baseline characteristics of excluded infants did not differ meaningfully from those included.

### 2.3. Data Collection

Data were extracted from a structured questionnaire completed at admission and updated daily throughout hospitalization. Maternal and perinatal variables included:

- *GBS* screening status;
- Duration of membrane rupture;
- Intrapartum fever >38 °C;
- Clinical or histological chorioamnionitis;
- Mode of delivery.

Neonatal variables included:

- Sex, gestational age, birth weight;
- Birth and admission dates;
- Transfer origin;
- Age at admission.

Clinical and microbiological data included:

- Empirical and targeted antibiotic regimens;
- Daily documentation of clinical system involvement (respiratory, CNS, gastrointestinal, skin), based on predefined clinical criteria;
- Presence of incipient, advanced, or persistent sepsis (definitions below);
- Culture results from blood or other sterile sites;
- Clinical outcomes.

Operational definitions:

To ensure clarity and consistency, the following definitions were applied:

- Incipient sepsis: early, nonspecific clinical signs suggestive of evolving infection (e.g., mild respiratory distress, feeding intolerance, temperature instability).
- Advanced sepsis: presence of severe clinical signs at admission, including septic shock, marked respiratory failure, or multiorgan involvement.
- Persistent sepsis: ongoing systemic signs of infection on Day 7 despite appropriate empirical antibiotic therapy and in the absence of an alternative diagnosis.

NICU Admission Criteria

Admission criteria included respiratory distress, suspected sepsis, prematurity, hypoglycemia, perinatal asphyxia, and any condition requiring continuous monitoring or specialized neonatal care.

### 2.4. Statistical Analysis

Statistical analysis was performed using IBM SPSS Statistics 25.0 (SPSS Inc. *233 S. Wacker Drive, 11th Floor, Chicago, IL 60606, USA*). Descriptive statistics was used to determine the mean values and standard deviation. Independent sample *t*-test was used to determine the difference between continuous variables divided into 2 groups and one-way ANOVA for variables divided into >2 groups with Turkey’s post hoc analysis to interpret the results. Univariate analysis was performed using Pearson’s correlation (Supplementary Material, Section S1, Tables S1 and S2).

### 2.5. Ethical Approval

The study protocol was reviewed and approved by the Ethics Committee for Human Research of Venizeleio General Hospital of Crete (approval number 56, date: 23 April 2024). Informed consent was not required, as per institutional policy, because the study used anonymized retrospective clinical data.

## 3. Results

### 3.1. Study Population

During the four-year study period, 458 neonates were evaluated for suspected or confirmed sepsis in our NICU. A total of 297 infants met the inclusion criteria, having been admitted within the first week of life and possessing complete medical records. Most neonates (99.3%, 295/297) were admitted before 72 h of life, while only 0.7% (2/297) were admitted between 72 h and 7 days, consistent with the EOS definition applied during the study period [19]. The two infants admitted after 72 h presented with respiratory distress and feeding intolerance, respectively.

Of the included infants, 190 (64%) were male and 107 (36%) female. The majority were of Greek ethnicity (83.6%). Neonates were admitted from the hospital’s maternity unit (2.2%), other public hospitals (60.4%), private maternity units (37.4%), or directly from the community.

The mean gestational age was 33.8 ± 4.42 weeks (range 23–41 weeks). Cesarean section was the mode of delivery in 75.8% (225/297). Prolonged rupture of membranes (>18 h) occurred in 16.9% of pregnancies, 12.8% of mothers received intrapartum antibiotics ≥4 h before delivery, and 4.40% had intrapartum fever >38 °C and/or clinical chorioamnionitis.

Among the 297 neonates evaluated for EOS, 28 (9.4%) had culture-proven sepsis. The overall EOS evaluation rate during the study period was 11.7 per 1000 live births, and EOS-related mortality was 0.35 per 1000 live births.

### 3.2. Clinical Presentation

Clinical signs of sepsis were most prominent on Day 1 and declined over time. Respiratory involvement was the predominant localization throughout the first week. Table 1 summarizes the distribution of clinical signs and infection sites at Days 1, 3, and 7 post-admission.

### 3.3. Antibiotic Therapy and Clinical Outcomes

All neonates received empirical broad-spectrum antibiotics. The most common regimen was ampicillin plus gentamicin (93.3%), followed by ampicillin plus cefotaxime (5.7%). Three infants with skin involvement received empirical cloxacillin, and *Staphylococcus epidermidis* was isolated in all targeted-therapy cases. A complete list of pathogens isolated in culture-proven EOS is provided in Supplementary Table S1. The initial empirical regimen was continued in 93.9% of infants, while 5.7% required targeted therapy.

Most neonates (96.7%) were discharged in good clinical condition. Nine infants (3%) died due to sepsis and complications related to prematurity. The mean NICU length of stay was 27.8 ± 31.1 days (range 0–182 days). A total of 6.40% were transferred to another hospital for further management. Transferred infants could not be followed longitudinally, limiting outcome assessment.

### 3.4. Factors Associated with Sepsis Severity and Outcomes

#### 3.4.1. Factors Associated with Sepsis at Day 1

Multivariate analysis identified several independent predictors of sepsis on Day 1:

- Lower gestational age (*p* = 0.035);
- Prolonged rupture of membranes >18 h (*p* = 0.043).

Infants presenting with advanced sepsis at admission had a distinct risk profile characterized by:

- Lower birth weight (*p* = 0.001);
- Lower gestational age (*p* < 0.001);
- Older maternal age (*p* = 0.001);
- Absence of intrapartum antibiotics ≥4 h before delivery (*p* = 0.001).

A concurrent identifiable focus of clinical system involvement at Day 1 was strongly associated with:

- Prolonged rupture of membranes (*p* < 0.001);
- Maternal intrapartum fever >38 °C and/or chorioamnionitis (*p* = 0.016).

#### 3.4.2. Factors Associated with Persistent Sepsis at Day 7

Infants who continued to exhibit clinical signs of sepsis at Day 7 were significantly more premature (*p* = 0.008). Persistent sepsis was also associated with:

- Higher mortality risk (*p* < 0.001);
- Longer NICU stay (*p* = 0.001);
- Prolonged antibiotic therapy (*p* = 0.003).

These findings indicate that early disease severity strongly predicted a prolonged clinical course.

#### 3.4.3. Factors Associated with Adverse Outcomes

Total NICU length of stay was significantly influenced by:

- Lower birth weight (*p* < 0.001)
- Lower gestational age (*p* < 0.001)
- Referral from another hospital (*p* = 0.002)
- Presence of advanced sepsis at Day 1 (*p* = 0.002)

Neonates who died were significantly more likely to:

- Have been born by vaginal delivery (*p* = 0.011);
- Present with advanced sepsis at Day 1 (*p* < 0.001);
- Exhibit persistent sepsis through Day 7;
- Develop a new or additional clinical system involvement by Day 3 (*p* = 0.022).

**Figure 1:**
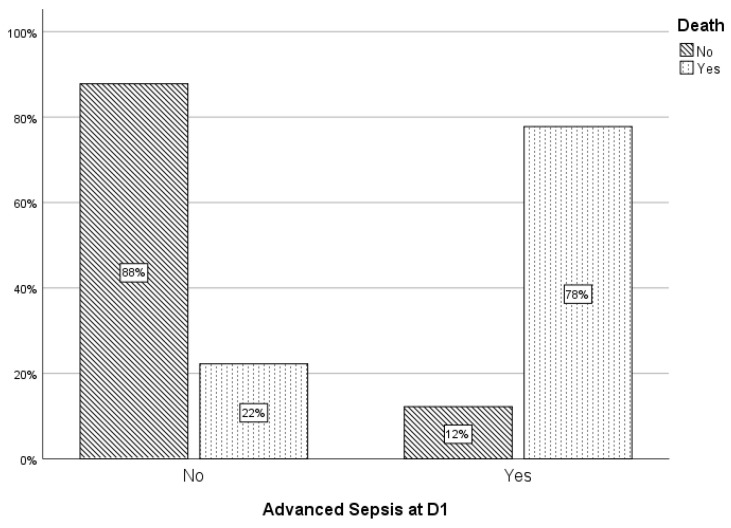
Correlation of advanced sepsis at Day1 with death.

**Figure 2:**
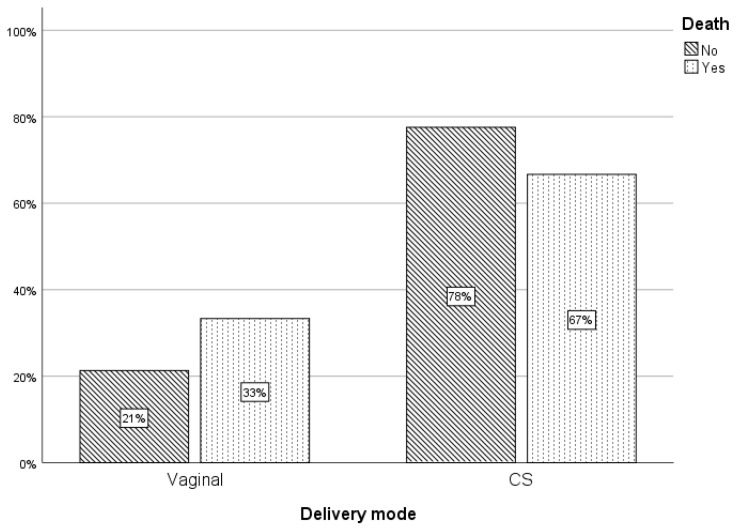
Percentage of death in relation to delivery mode.

Figure 1 provides a detailed breakdown of mortality among infants with advanced sepsis at admission, and Figure 2 illustrates the mortality distribution by mode of delivery.

## 4. Discussion

In this retrospective cohort, the respiratory system emerged as the most frequently affected clinical system on the first day of NICU admission in neonates evaluated for EOS. This finding aligns with the pathophysiology of early-onset disease, where perinatal respiratory adaptation, aspiration of infected amniotic fluid, and immature pulmonary immune defenses contribute to early respiratory manifestations [20,21]. Importantly, respiratory involvement in our study reflects clinical signs rather than microbiologically confirmed pulmonary infection. Central nervous system, gastrointestinal, and skin involvement were less commonly observed at presentation, consistent with the literature indicating that these manifestations typically arise later in the disease course or in association with specific risk factors [22,23,24].

To our knowledge, this is the first study to systematically map the evolution of clinical system involvement during the first week of life using prospectively documented daily assessments. By capturing dynamic changes rather than relying solely on admission findings, our study provides novel insight into early disease trajectories in EOS. The appearance of a new or additional clinical system involvement by Day 3 was strongly associated with mortality, underscoring the prognostic value of early clinical progression.

Our findings reinforce the well-established association between prematurity, low birth weight, and increased vulnerability to severe neonatal sepsis [8,25,26]. Premature infants lack adequate transplacental transfer of maternal IgG, which accelerates during the third trimester, rendering them more susceptible to invasive infections. Emerging evidence also suggests that low 25-hydroxyvitamin D levels in preterm neonates may further compromise immune function, supporting recommendations for maternal vitamin D supplementation during pregnancy [26,27,28,29].

Maternal intrapartum factors—including prolonged rupture of membranes and intrapartum fever—were strongly associated with early clinical system involvement, consistent with previous studies demonstrating increased sepsis risk under these conditions [4,30]. The maternal urogenital tract remains a major reservoir for pathogens implicated in EOS, and prolonged membrane rupture facilitates ascending infection and fetal exposure.

Local microbial epidemiology in Crete largely mirrors national and European patterns, with *Group B Streptococcus* and *Escherichia coli* predominating in EOS. However, surveillance data indicate a higher contribution of Gram-negative pathogens in Greece, including *Klebsiella* spp. and *Enterobacteriaceae*, which may influence empirical therapy choices in different regions. We also provide, for the first time, a detailed list of pathogens isolated in culture-proven EOS within our cohort, allowing comparison with international settings where *GBS* screening protocols and antimicrobial resistance patterns differ (Supplementary material, Section S2).

Empirical antibiotic therapy in our cohort followed international recommendations, with most neonates receiving ampicillin plus gentamicin. This regimen proved effective, as the majority of infants demonstrated clinical improvement [25,31,32]. In cases where *Staphylococcus epidermidis* was isolated, targeted therapy with cloxacillin was appropriate, although susceptibility patterns vary and culture-guided therapy remains essential [33,34].

A key distinction in our study is the differentiation between culture-proven and clinical EOS. Only 9.4% of cases were culture-confirmed, consistent with global reports of low culture positivity due to intrapartum antibiotic exposure, low blood culture volumes, and early sampling. This distinction represents an important limitation of the study.

This study has several strengths. It includes a relatively large cohort of neonates evaluated for EOS over a four-year period, providing a robust dataset for identifying clinically meaningful associations. The use of a structured, prospectively completed daily questionnaire ensured systematic documentation of maternal, perinatal, and neonatal variables. Daily clinical updates allowed for precise characterization of disease progression, representing a methodological strength of the study.

Several limitations should also be acknowledged. The retrospective design restricts the completeness of available data, and missing documentation led to the exclusion of 161 cases. Although the excluded infants did not differ meaningfully in baseline characteristics, this may have introduced selection bias. Additionally, neonates transferred to other hospitals could not be followed longitudinally, limiting assessment of their full clinical course and outcomes. As a single-center study, generalizability may be limited, particularly in settings with different microbial epidemiology, obstetric practices, or NICU protocols. Finally, certain subgroups—such as infants with specific clinical system involvement or advanced sepsis—were relatively small, reducing statistical power.

## 5. Conclusions

This study highlights the substantial burden of early-onset neonatal sepsis and reinforces the importance of established risk factors such as prematurity, low birth weight, prolonged rupture of membranes, and maternal intrapartum infection. Respiratory involvement was the most common clinical system affected at admission, emphasizing the need for early respiratory evaluation in neonates with suspected EOS. Mapping the evolution of clinical signs across the first week of life provides valuable insight into early disease trajectories and identifies neonates at risk for persistent sepsis and adverse outcomes. Continued efforts to refine risk stratification, optimize empirical antibiotic use, and strengthen antibiotic stewardship programs are essential. Multicenter prospective studies are warranted to further elucidate pathogen patterns, refine predictive models, and guide individualized management strategies for neonatal sepsis.

## Figures and Tables

**Table 1:** Neonates with incipient sepsis signs at three post-admission time points and pathogen localization sites. CNS: central nervous system, GI: gastrointestinal tract.

	Day 1	Day 3	Day 7
Incipient sepsis signs	89.2%	50.2% (persistent)	6.5% (persistent)
Respiratory tract infection	57.2%	58%	24.5%
CNS involvement	5%	0%	0%
GI tract involvement	2%	0%	0%
Skin involvement	1.6%	0%	0%

## Data Availability

Data available upon reasonable request.

## Supplementary Materials

The following supporting information can be downloaded at: https://www.mdpi.com/article/10.3390/children13050639/s1, Supplementary Materials, Section S1: Table S1 Patient data, Table S2 Descriptive statistics, Section S2: Full list of all pathogens isolated in culture-proven EOS.
