# Supplementary material for: Early-Onset Neonatal Sepsis: Clinical System Involvement and Maternal–Neonatal Risk Profiles in a Retrospective Cohort Study
Source: Children (Basel). 2026 May 3;13(5):639. doi: 10.3390/children13050639 (PMC13204373; doi:10.3390/children13050639)
Supplement: Supplementary file 1 [file children-13-00639-s001.zip › children-4260307-supplementary.pdf]

Supplementary material, Section S1

Table S1: Patient data

	Male	Female	Total (N)
**Newborn sex**	190 (64%)	107 (36%)	297
	**Greek**	**Other**	**Total (N)**
**Ethnicity**	235 (83.6%)	46 (16.4%)	281
	**Vaginal**	**Cesarian section**	**Total (N)**
**Birth**	69 (23.2%)	225 (75.8%)	297
	**Yes**	**No**	**Total (N)**
**Newborn age <72h**	295 (99.3%)	2 (0.7%)	297
**Membranes rupture >18h**	50 (16.9%)	236 (79.7%)	286
**Advanced Sepsis at day 1**	41 (13.9%)	255 (86.1%)	296
**Advanced Sepsis at day 7**	1 (0.6%)	155 (99.4%)	156
**Fever >38oC/ Chorioamnionotis**	13 (4.4%)	280 (94.3%)	293
**Transfer from other hospitals**	19 (7.4%)	239 (92.6%)	258
**Death**	9 (3.3%)	263 (95.6%)	275

Table S2: Descriptive statistics

	Minimum	Maximum	Mean	Std. Deviation	
**BirthWeight (g)**	470	4700	2289,90	939,310	
**GestationalWeek**	23	41	33,84	4,423	
**NewbornAge (days)**	0	303	2,78	24,531	
**Total stay in NICU (days)**	0	394	30,42	44,963	

Supplementary Material, Section S2

Full list of all pathogens isolated in culture-proven EOS

- Acinetobacter baumannii
- Bacillus circulans
- Burkholderia cepacia
- Candida albicans
- Candida lusitaniae
- Candida pelliculosa
- Citrobacter koseri
- Enterobacter aerogenes
- Enterobacter cloacae
- Enterococcus faecalis
- Escherichia coli (E. coli)
- Group B Streptococcus (GBS)
- Klebsiella oxytoca
- Klebsiella pneumoniae
- Listeria (usually Listeria monocytogenes)
- Microbacterium species
- Prevotella bivia
- Pseudomonas aeruginosa
- Serratia marcescens
- Staphylococcus aureus
- Staphylococcus epidermidis
- Staphylococcus hominis
- Staphylococcus lugdunensis
- Staphylococcus warneri
- Streptococcus haemolyticus
